# Upregulation of *Pnpla2* and *Abhd5* and downregulation of *G0s2* gene expression in mesenteric white adipose tissue as a potential reason for elevated concentration of circulating NEFA after removal of retroperitoneal, epididymal, and inguinal adipose tissue

**DOI:** 10.1007/s11010-016-2800-4

**Published:** 2016-09-02

**Authors:** Agnieszka Dettlaff-Pokora, Tomasz Sledzinski, Julian Swierczynski

**Affiliations:** 1 Department of Biochemistry, Medical University of Gdansk, Dębinki 1, 80-211 Gdansk, Poland; 2 Department of Pharmaceutical Biochemistry, Medical University of Gdansk, Dębinki 1, 80-211 Gdansk, Poland

**Keywords:** ATGL, G0S2, ABHD5, HSL, NEFA, Lipolysis, Adipose tissue

## Abstract

Elevated concentrations of circulating non-esterified fatty acids (NEFA) were reported in (a) humans with lipodystrophy, (b) humans following bariatric surgery, and (c) transgenic mice with reduced amounts of adipose tissue. Paradoxically, these findings suggest that the reduction of adipose tissue mass is associated with elevated circulating NEFA concentrations. To explain a molecular background of this phenomenon, we analyzed the effects of surgical removal of inguinal, epididymal, and retroperitoneal white adipose tissue (WAT) on (a) circulating NEFA concentrations, (b) expression of *Pnpla2*, a gene that encodes adipose triglyceride lipase (ATGL), genes encoding abhydrolase domain containing 5 (ABHD5) and G0/G1 switch 2 (G0S2), i.e., a coactivator and inhibitor of ATGL, respectively, and (c) expression of *Lipe* gene coding hormone-sensitive lipase (HSL) in mesenteric WAT. Reduction of adipose tissue mass resulted in an increase in circulating NEFA concentration, which was associated with (a) an increase in the expressions of *Pnpla2* and *Abhd5*, (b) decrease in *G0s2* expression, and (c) upregulation of *Lipe* expression, all measured on both mRNA and protein levels in mesenteric WAT of male rats. The rate of lipolysis in mesenteric WAT explants and isolated adipocytes from lipectomized rats was significantly higher than that from the controls. In conclusion, upregulation of *Pnpla2* expression and activation of ATGL (due to an increase in ABHD5 and decrease in G0S2 levels), as well as a coordinated interplay of these genes with *Lipe* in mesenteric WAT, contribute, at least in part, to an increase in the concentration of circulating NEFA in rats with reduced fat mass.

## Introduction

Excess of adipose tissue (e.g., in obese subjects) is generally believed to be associated with increased risk of metabolic syndrome, insulin resistance, and diabetes mellitus [1–4]. These abnormalities are usually associated with an increase in circulating NEFA concentration and consequently, with accumulation of ectopic fat, which may play an important role in the pathogenesis of non-insulin-dependent diabetes mellitus [3, 4], lipotoxicity [5–7], and inflammation [5, 7]. Paradoxically, complete lack or deficiency of adipose tissue, as seen in lipodystrophy and lipoatrophy, is also associated with an increase in the concentration of circulating NEFA [8], greater risk of metabolic syndrome, insulin resistance, diabetes mellitus, and cardiovascular diseases [9–13]. These abnormalities result, at least in part, from accumulation of ectopic triacylglycerols in the liver and muscles, rather than in adipocytes [14]. Elevated levels of circulating NEFA were also observed in transgenic mice (A-ZIP/F-1 mice) deprived of white adipose tissue (WAT) [15]. Altogether, these data imply that the deficiency of adipose tissue may be associated with elevated concentration of circulating NEFA, in both animals and humans. However, a molecular background of the increase in circulating NEFA concentration in patients with lipodystrophy and rats with adipose tissue deficiency is still not understood.

Recently, Ling et al. [16] demonstrated that partial lipectomy results in upregulated expression of *Lipe* gene (encoding hormone-sensitive lipase, HSL) in rat liver, and suggested that this may contribute to excessive accumulation of NEFA. Concentration of circulating NEFA depends, among other factors, on the rate of lipolysis in adipose tissue. Lipolysis is initiated by adipose triglyceride lipase (ATGL, also referred to as desnutrin, TTS2.2, and iPLA_2_ ζ, encoded by *Pnpla2* gene). Activity of ATGL is modulated by abhydrolase domain containing 5 (ABHD5, a coactivator of ATGL activity, encoded by *Abhd5* gene) and G0/G1 switch 2 (G0S2, an inhibitor of ATGL activity, encoded by *G0s2* gene) [17–19]. However, little is known on the regulation of ATGL-encoding genes and proteins that control the ATGL activity (ABHD5 and G0S2) after reduction of adipose tissue. We hypothesized that lipectomy is reflected not only by enhanced expression of *Lipe* in the liver [16] but also by an increase in the expressions of *Pnpla2, Abhd5*, and *Lipe* in WAT, as well as by a downregulation of *G0s2* gene, which altogether lead to an increase in the concentration of circulating NEFA.

The aim of this study was to determine the concentration of circulating NEFA and the expressions of *Pnpla2, Lipe, Abhd5*, and *G0s2* genes (the latter two encoding proteins involved in the regulation of ATGL) in mesenteric WAT, following significant reduction of epididymal and retroperitoneal WAT mass and total removal of inguinal WAT. Our observations imply that post-lipectomy status, which likely mimics human lipodystrophy, lipoatrophy, or state following bariatric surgery, may be linked to upregulation of *Pnpla2, Abhd5*, and *Lipe* genes and downregulation of *G0s2* gene in mesenteric WAT of rats. These changes are associated with enhanced in vitro lipolysis and with an increase in the concentration of circulating NEFA.

## Materials and methods

### Animals and surgery

The study rats were fed with a commercial diet [20] and treated as previously described [21, 22]. Briefly, 12-week-old male Wistar rats were randomly divided into two groups (*n* = 10 each): (1) lipectomized animals that were subjected to surgical resection of epididymal and retroperitoneal WAT, and (2) control animals that were anesthetized and subjected to a sham procedure (incision of the skin and muscles without removal of WAT). One month after the first surgery, the lipectomized rats were anesthetized again to remove their subcutaneous (inguinal) WAT, while the controls were subjected to another sham surgery. We decided to perform lipectomy as the two-step procedure in order to reduce perioperative mortality. The surgeries were conducted carefully to avoid bleeding. Mean weight of fat removed from the lipectomized rats was 7.7 ± 0.6 g (3.8 ± 0.3, 2.0 ± 0.4 and 1.9 ± 0.4 g for subcutaneous, epididymal, and retroperitoneal WAT, respectively). Three months after the first surgery, the animals were anesthetized and killed by decapitation (between 8:00 a.m. and 10:00 a.m.). All the procedures related to animal handling and care were approved by the Local Bioethics Committee at the Medical University of Gdansk. Blood samples from the carotid artery were collected into tubes without anticoagulant and centrifuged at 3000×*g* for 15 min at 4 °C to obtain sera that were stored at −20 °C until analysis. Upon removal, the samples of mesenteric fat were weighed and immediately thereafter frozen in liquid nitrogen. Also the samples of the liver, renal cortex and skeletal muscles (the latter deprived of any visible deposits of fat) were immediately frozen in liquid nitrogen. All the tissue specimens were stored at −80 °C until analysis. Epididymal, retroperitoneal, and subcutaneous WAT from the controls, as well as a residual WAT from the lipectomized animals, were removed and weighed.

### Isolation of RNA and determination of mRNA level

Total cellular RNA was isolated from frozen samples of mesenteric WAT with guanidinium isothiocyanate/phenol/chloroform method [23]. Concentration of RNA was determined on the basis of absorbance at 260 nm; all the samples showed a 260/280 nm absorbance ratio of about 2.0. Prior to the reverse transcription, the samples of RNA were treated with RNase-free DNase I (Fermentas International, Inc., Canada). First-strand cDNA synthesis and determination of mRNA levels by means of RT-PCR were performed as described previously [21], using the Chromo4 real-time detection system (Bio-Rad Laboratories, Inc., USA). The sequences of primers used in this study are listed in Table 1. β-actin mRNA was used as an internal standard. Relative quantities of the transcripts were calculated from the 2^−ΔΔCT^ formula [24]. Amplification of specific transcripts was further confirmed on the basis of their melting-curve profiles.

**Table 1:** Primer sequences used in this study

Gene	Primer sequence (5′-3′)
*Lipe*	F:AAT GAC ACA GTC GCT GGT GGC G
	R:TGC CAC ACC CAA GAG CTG ACC T
*Pnpla2*	F:CCC TGA CTC GAG TTT CGG AT
	R:CAC ATA GCG CAC CCC TTG AA
*Abhd5*	F:AAC CCC AAG TGG TGA GAC AG
	R:GCG CCG AAG ATG ACT GAA AC
*G0s2*	F:TGA CCT CCT TCA GCG AGT G
	R:TCG GGA CTT CTG CGT CAT C
*Actb*	F:TGT CAC CAA CTG ACG ATA
	R:GGG GTG TTG AAG GTC TCA AA

### Lipolysis assay in adipose tissue explants

Lipolysis assay in explants of mesenteric adipose tissue was performed as described recently [25]. Lipolysis was stimulated with forskolin (final concentration of 10 µM) and dibutyryl-cAMP (final concentration of 0.2 mM).

### Isolation of adipocytes and lipolysis assay in isolated adipocytes

Adipocytes were isolated as described previously [26], with few modifications. The fragments of mesenteric fat for adipocyte isolation were collected and washed in warmed (37 °C) Krebs–Ringer–Henseleit buffer containing 5.5 mM of glucose, 10 mg/ml of bovine serum albumin (fatty acid free), and 20 mM of Hepes/Na (pH 7.4) (all chemicals provided by Sigma Aldrich). The fragments of fat were cut into small pieces and incubated in Krebs–Ringer–Henseleit buffer enriched with 1 mg/ml collagenase (*Clostridium histolyticum* type II collagenase from Sigma) for 1 h at 37 °C, with gentle shaking. Then, the material was filtered through 180-µm nylon filters (Millipore), and the adipocytes that have been collected on the filters were rinsed three times with Krebs–Ringer–Henseleit buffer. Mesenteric adipocytes from each rat were divided into three 0.2-ml cell suspension aliquots in 1.8 mL of Krebs–Ringer–Henseleit buffer and subjected to lipolysis as described recently [25]. Lipolysis was stimulated with forskolin (final concentration of 10 µM) and dibutyryl-cAMP (final concentration of 0.2 mM).

### Carnitine palmitoyl transferase 1 (CPT1) activity assessment

Liver, renal cortex, and skeletal muscle samples were minced and homogenized in a glass homogenizer in a 1:10 (wt/vol) dilution in buffer containing 250 mM of sucrose, 0.01 M of Tris, and 0.5 mM of EDTA (pH 7.4). Then, the homogenates were centrifuged at 4 °C for 3 min at 300×*g* (skeletal muscles and renal cortex) or for 10 min at 600×*g* (liver). Activity of CPT1 and content of the protein were determined in supernatant, as described by Bieber et al. [27] and Lowry et al. [28], respectively.

### Mitochondrial palmitoyl-carnitine oxidation

Mitochondria from liver, kidney cortex, and skeletal muscles were isolated as described by Bremer and Norum [29] and Swierczynski et al. [30]. Palmitoyl-carnitine oxidation was measured as described previously [31].

### Determination of NEFA concentration

Serum concentration of NEFA, as well as the levels of NEFA released by mesenteric adipose tissue or isolated adipocytes, was measured with an enzymatic colorimetric method according to the protocol supplied by Wako Chemicals GmbH (Germany).

### Determination of glycerol concentration

Concentrations of glycerol released by mesenteric adipose tissue and isolated adipocytes were measured with an enzymatic colorimetric method according to the protocol supplied by BioVission (Milpitas, CA 95035, USA).

### SDS-PAGE and immunoblotting

Frozen specimens of mesenteric fat were homogenized in 20 mM of Tris–HCl buffer (pH 7.8) containing 0.2 % of Triton X-100 and protease inhibitor cocktail (Sigma, USA), and centrifuged. Aliquots of supernatant (10 μg of protein) were separated by 10 % of SDS-PAGE and electroblotted onto Immuno-Blot™ PVDF Membrane (Bio-Rad Laboratories, Hercules, CA, USA). The membrane was blocked by incubation with a blocking buffer and then incubated with rabbit polyclonal anti-HSL antibody (SCB45041763, Sigma-Aldrich USA), polyclonal anti-phospho-HSL antibody (pSer^522^ in human/Ser^563^ in rat; SAB4501763, Sigma-Aldrich USA), anti-ABHD5 antibody (AV42055, Sigma-Aldrich USA), anti-ATGL antibody (sc-67355, Santa Cruz Biotechnology USA), anti-G0S2 antibody (sc-133424, Santa Cruz Biotechnology USA), and anti-actin antibody (A0545, Sigma-Aldrich USA). The secondary HRP-conjugated antibodies were obtained from Sigma Aldrich (A0545). The reactions were visualized with SuperSignal West Pico chemiluminescent substrate (Thermo Fisher Scientific, Inc., Rockford, IL, USA). The bands visualized on the film following chemiluminescent detection were compared with the molecular mass protein markers visible on the membrane after electroblotting (SM26634, Fermentas). The film was adjusted to the membrane in such way that the membrane edges were visible on the film.

### Statistical analysis

Statistical calculations were carried out with an Excel 2010 spreadsheet (Microsoft). All results for the controls and lipectomized rats are expressed as mean values (±SD). The significance of intergroup differences in the analyzed parameters was verified with Student’s *t* test. The differences were considered significant at *p*-value <0.05.

## Results

Initial body masses of the control and lipectomized rats were essentially similar (Table 2). Three months after the first surgery, body mass of lipectomized rat increased significantly. The increase in body mass of lipectomized rats was essentially similar that of control rats (Table 2). Lipectomy resulted in complete removal of inguinal adipose tissue, and the weight of retroperitoneal and epididymal adipose tissue in lipectomized rats was ca. 80 % lower than in the controls (Table 2). Altogether, this corresponded to approximately 90 % reduction of the overall adipose tissue content in these three fat residues (Table 2). However, lipectomized rats presented with significantly greater mass of mesenteric adipose tissue and liver than the controls (Table 2).

**Table 2:** Body mass, adipose tissue mass, and liver mass in the lipectomized and control rats

	Control	Lipectomy
Initial body weight (g)	312 ± 18	315 ± 19
Mesenteric fat (g)	5.1 ± 0.1	6.3 ± 0.2*
Weight of removed fat (total) (g)	–	7.7 ± 0.6
Final body weight (g)	403 ± 21	407 ± 17
Final total fat (g)	15.0 ± 3.9	1.5 ± 0.9*
Epididymal fat (g)	5.4 ± 1.6	0.7 ± 0.5*
Retroperitoneal fat (g)	4.7 ± 1.9	0.8 ± 0.5*
Inguinal fat (g)	4.9 ± 1.2	0*
Liver (g)	16.0 ± 0.7	18.0 ± 0.9*

*p < 0.05

**Fig. 1:**
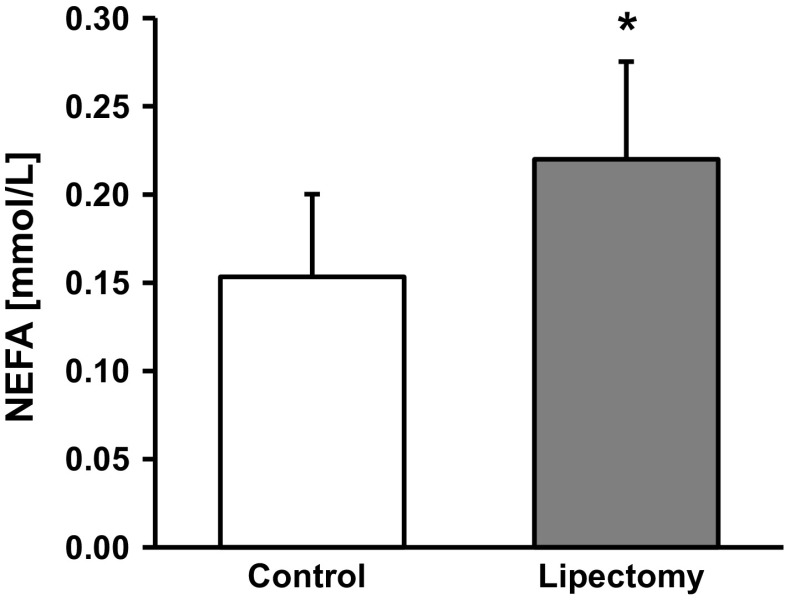
Serum concentrations of non-esterified fatty acids (NEFA) in the controls and lipectomized rats. Data are presented as mean ± SD. *p < 0.05

**Fig. 2:**
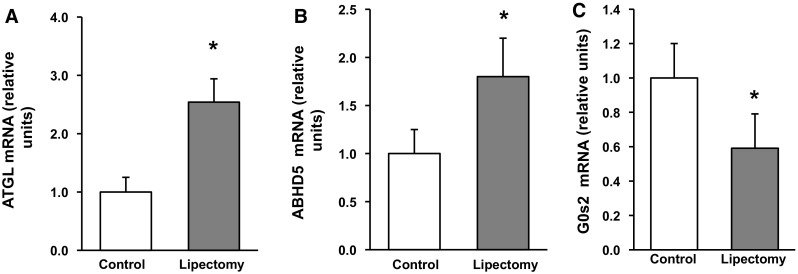
Relative mRNA levels of **A** adipose triglyceride lipase (ATGL), **B** abhydrolase domain containing 5 (ABHD5), and **C** G0/G1 switch 2 (G0S2) in mesenteric adipose tissue of the controls and lipectomized rats. Data are presented as mean ± SD. *p < 0.05

**Fig. 3:**
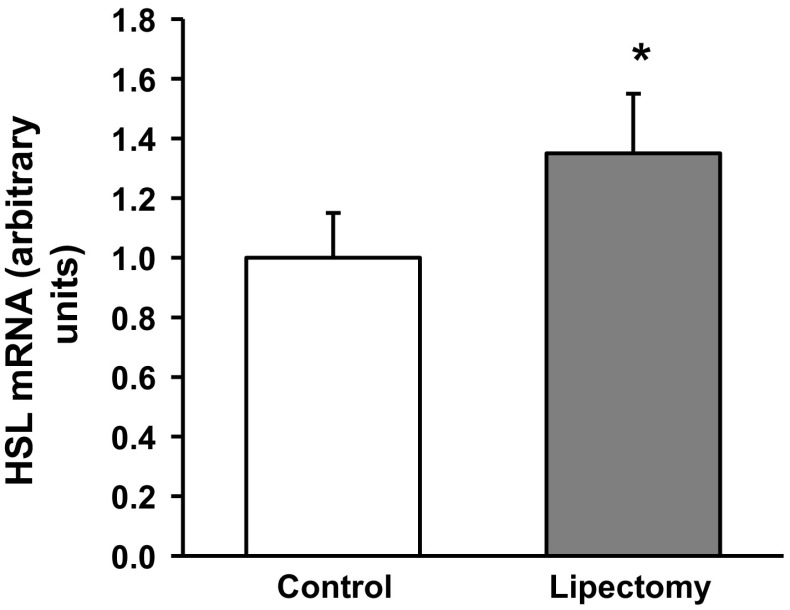
Relative mRNA levels for hormone-sensitive lipase (HSL) in mesenteric adipose tissue of the controls and lipectomized rats. Data are presented as mean ± SD. *p < 0.05

Partial lipectomy was reflected by approximately 50 % increase in the serum concentration of NEFA (Fig. 1). Moreover, mesenteric WAT from lipectomized rats contained higher levels of ATGL mRNA (encoded by *Pnpla2)* (Fig. 2A). Partial lipectomy turned out to be also associated with an increase in ABHD5 (also referred to as CG1-58) mRNA level in mesenteric WAT (Fig. 2B) and with a significant decrease in WAT G0S2 mRNA level in mesenteric WAT (Fig. 2C). Finally, partial lipectomy was shown to result in an increase in HSL (Fig. 3) mRNA level in mesenteric adipose tissue.

**Fig. 4:**
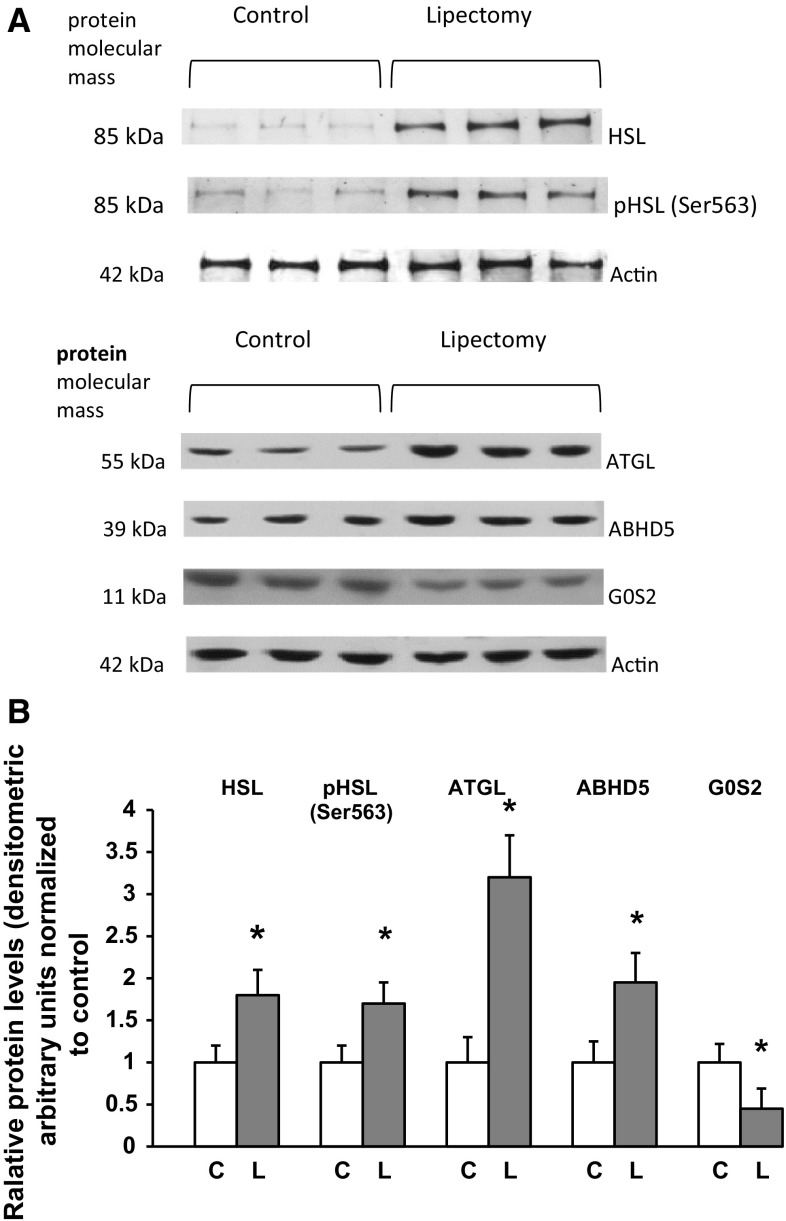
Western blot analysis of hormone-sensitive lipase (HSL, non-phosphorylated and phosphorylated-Ser^563^), adipose triglyceride lipase (ATGL), abhydrolase domain containing 5 (ABHD5) and G0/G1 switch 2 (G0S2) from mesenteric adipose tissue of the controls (C) and lipectomized rats (L). **A** Representative western blot analysis of ATGL, ABHD5, G0S2, HSL, and phosphorylated HSL (Ser^563^) standardized against actin in mesenteric adipose tissue of the controls and lipectomized rats; **B** densitometric analysis of western blot bands. Data are presented as mean ± SD. *p < 0.05

Western blot analysis showed that the abovementioned intergroup differences in mRNA levels (Figs. 2, 3) were reflected by different levels of HSL (both non-phosphorylated and phosphorylated forms), ATGL, ABHD5 (a significant post-lipectomy increase), and G0S2 (a significant post-lipectomy decrease) proteins in the controls and lipectomized rats (Fig. 4A representative western blots, Fig. 4B densitometric analysis).

**Fig. 5:**
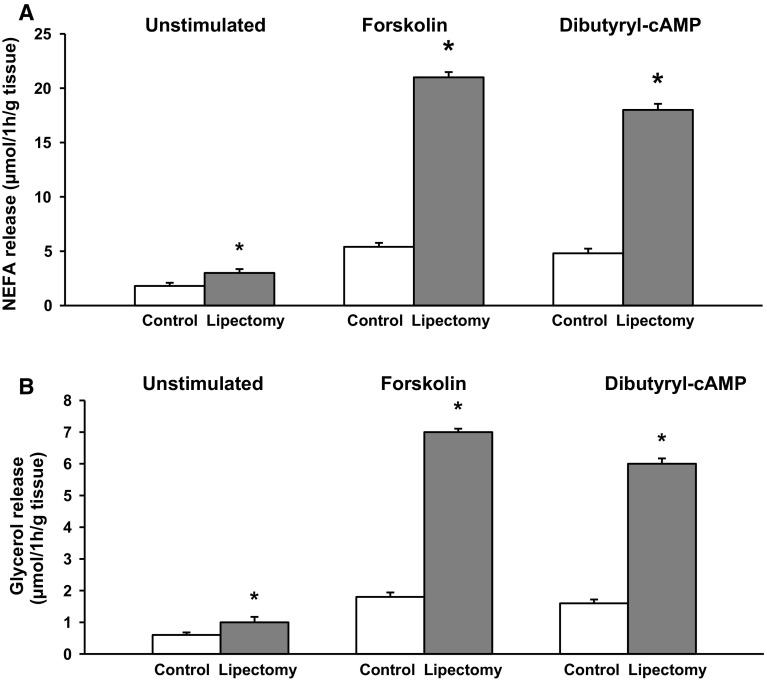
The rate of lipolysis in mesenteric adipose tissue explants from the controls and lipectomized rats. The graph depicts unstimulated and stimulated release of NEFA (**A**) and glycerol (**B**) from mesenteric adipose tissue. Lipolysis was stimulated with forskolin (final concentration of 10 µM) and dibutyryl-cAMP (final concentration of 0.2 mM). The results are presented as mean ± SD. *p < 0.05

**Fig. 6:**
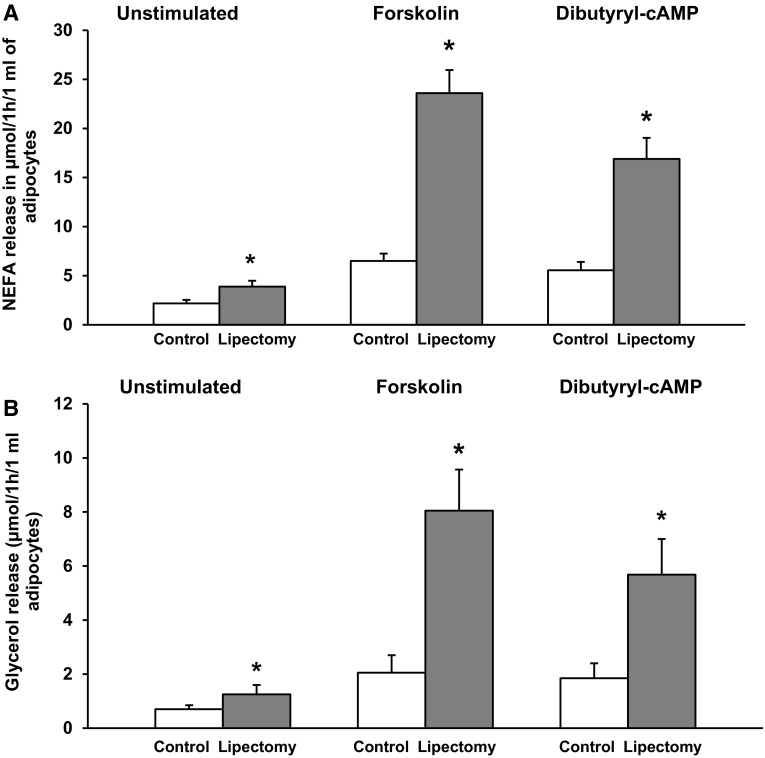
The rate of lipolysis in isolated adipocytes of mesenteric adipose tissue from the controls and lipectomized rats. The graph depicts unstimulated and stimulated release of NEFA (**A**) and glycerol (**B**) from mesenteric adipose tissue. Lipolysis was stimulated with forskolin (final concentration of 10 µM) and dibutyryl-cAMP (final concentration of 0.2 mM). The results are presented as mean ± SD. *p < 0.05

To verify if the abovementioned changes at mRNA and protein level were truly reflected by an increase in the concentration of circulating NEFA, we determined the rate of lipolysis in mesenteric WAT explants from the controls and partially lipectomized rats. The rate of unstimulated (i.e., measured in absence of dibutyryl-cAMP or forskolin) and stimulated (i.e., measured in presence of dibutyryl-cAMP or forskolin) lipolysis, determined in vitro based on NEFA (Fig. 5A) and glycerol (Fig. 5B) release from the explants, turned out to be significantly higher in lipectomized rats than in the controls. Essentially similar results were obtained for adipocytes isolated from mesenteric WAT (Fig. 6A, B). Consequently, the results presented in Figs. 5 and 6 document a cause–effect relationship between the enhanced lipolysis in mesenteric WAT and the increase in circulating NEFA concentration in lipectomized rats.

Theoretically, the increase in the concentration of circulating NEFA might also result from decreased uptake of the latter and their oxidation in various organs including liver, kidneys, and skeletal muscles. To verify this hypothesis, we determined the activity of CPT1 in the liver, renal cortex, and skeletal muscle extracts from the controls and lipectomized rats, as well as palmitoyl-carnitine (+malate) oxidation by mitochondria isolated from these organs. The results imply that lipectomy did not affect significantly either the CPT1 activity or the palmitoyl-carnitine (+malate) oxidation in the liver, renal cortex, and skeletal muscles (data not shown).

## Discussion

The data presented in this paper indicate that following surgical reduction of epididymal and retroperitoneal WAT mass and total surgical removal of inguinal WAT, circulating NEFA concentration significantly increases. It is generally accepted that most circulating NEFA concentrations originate from lipolysis in adipose tissue. Thus, it is likely that following lipectomy in rats, which may mimic (as far as reduction of fat mass and an increase in circulating NEFA concentration is concern) human lipodystrophy, lipoatrophy or state following bariatric surgery, in remaining WAT (mesenteric WAT), an increase in lipolysis will occur. The data presented in this paper suggest that this is the case. The novel and important finding of this study is that partial lipectomy (i.e., removal of retroperitoneal, epididymal, and inguinal WAT) is associated with an increase in *Pnpla2* gene expression in rat mesenteric WAT. ATGL is known to play a key role in the regulation of lipolysis in adipocytes [17–19, 32]; consequently, enhanced expression of *Pnpla2* in mesenteric adipose tissue seems to contribute, at least in part, to an increase in the concentration of circulating NEFA in lipectomized rats. This hypothesis is also supported by a positive correlation between the expression of *Pnpla2* in mesenteric WAT and the concentration of circulating NEFA (*r* = 0.66, *p* < 0.05).

The rate of lipolysis is also modulated by complex interactions between ATGL, ABHD5 (a coactivator of ATGL activity), and G0S2 (an inhibitor of ATGL activity) [17–19]. Recent findings suggest that G0S2 may exert strong effects on lipolysis, TAG deposition, and energy metabolism [33, 34]. In line with these data, our present study showed for the first time that the expressions of *Abhd5* and *G0s2* in mesenteric WAT may be also subjected to post-lipectomy upregulation and downregulation, respectively. The fact that we observed a post-lipectomy upregulation of *Pnpla2 and Abhd5* genes and a downregulation of *G0s2* gene in mesenteric WAT, as well as recent evidence suggesting that G0S2 may exert strong effects on lipolysis, TAG deposition and energy metabolism [33, 34], altogether imply that ATGL may be involved in a lipectomy-mediated increase in the concentration of circulating NEFA.

Ling et al. [16] demonstrated that partial lipectomy results in the upregulation of *Lipe* gene in rat liver. Our hereby presented findings imply that the *Lipe* gene may be also upregulated in mesenteric WAT of lipectomized rats (Figs. 3, 4). This suggests that the effect of lipectomy on *Pnpla2, Abhd5*, *G0s2*, and *Lipe* gene expressions in mesenteric WAT may serve as an example of long-term coordinated upregulation of adipose tissue lipolysis. This conclusion is also supported by the fact that our lipectomized rats presented with enhanced lipolysis, as shown by a greater release of NEFA and glycerol from the explants of mesenteric WAT (Fig. 5) and isolated adipocytes from mesenteric WAT (Fig. 6).

Consequently, it is excessive lipolysis which most likely contributes to an increase in the concentration of circulating NEFA; the latter, in turn, may attenuate the effects of insulin on its target organs. This may explain, at least in part, why complete lack of adipose tissue or deficiency thereof, as seen in lipodystrophy, are associated with an increase in the concentration of circulating NEFA and resultant elevated risk of metabolic syndrome, insulin resistance, diabetes mellitus, and cardiovascular diseases [9–13].

Consequently, a key question arises about the most likely reasons behind the upregulation of *Pnpla2, Abhd5*, and *Lipe* genes and downregulation of *G0s2* gene in mesenteric WAT of lipectomized rats. Noticeably, similar changes in the expression of lipolytic genes are also observed during fasting [32]. Thus, one may hypothesize that the upregulation of *Pnpla2, Abhd5*, and *Lipe* genes and downregulation of *G0s2* in mesenteric WAT of lipectomized rats represent a response to the shortage of energy reservoir resulting from removal of adipose tissue. One may suppose that it is insulin resistance which contributes primarily to the enhanced lipolysis observed in lipectomized rats. However, the factor(s) that lead to an increase in the rate of lipolysis in lipectomized rats are still not fully understood.

It has been proposed previously that due to storage of triacylglycerols, adipose tissue acts as a buffer, suppressing the release of NEFA into circulation and enhancing the clearance of triacylglycerols during postprandial period [35]. Consequently, the volume of adipose tissue left after lipectomy may be insufficient to uptake NEFA that have been released from VLDL-TAGs during postprandial period, and to metabolize them to adipose tissue TAGs. This may be another reason behind the increase in the concentration of circulating NEFA in lipectomized rats.

Adipose tissue and liver are known to cooperate in the maintenance of systemic energy homeostasis in response to food intake and deprivation thereof. In a fed state, both the liver and adipose tissue synthesize TAGs that are then stored in the latter. In turn, fasting is associated with enhanced lipolysis within WAT and diminished lipogenesis in the liver. Recent findings published by Ling et al. [16] and Dettlaff-Pokora et al. [22] indicate that lipectomy may enhance lipogenesis in rat liver (similar to a fed state), and the results of our present study imply that this procedure may also increase the rate of lipolysis within adipose tissue (as during fasting). Consequently, lipectomy may disrupt cooperation of liver and adipose tissue in the maintenance of systemic energy homeostasis due to reduction of energy reservoir.

Although the hereby presented experimental model does not necessarily replicate lipodystrophy, lipoatrophy, and state after bariatric surgery, it likely reflects all key metabolic changes observed in humans with these conditions. Consequently, it seems to be useful for research on molecular mechanisms that contribute to the increase in circulating NEFA concentration in subjects with reduced mass of adipose tissue.

In conclusion, this study showed that partial surgical removal of epididymal and retroperitoneal and total removal of inguinal fat is associated with upregulation of *Pnpal2, Abhd5*, and *Lipe* genes and downregulation of *G0s2* gene in rat mesenteric WAT. These changes coexist with enhanced lipolysis in mesenteric WAT explants and isolated adipocytes, as well as with an increase in the concentration of circulating NEFA. Consequently, the post-lipectomy increase in circulating NEFA concentration seems to result, at least in part, from enhanced lipolysis within the remaining adipose tissue. These findings add to existing knowledge on changes in TAG breakdown within mesenteric adipose tissue that occur in response to partial lipectomy which likely mimics human lipodystrophy, lipoatrophy, or state following bariatric surgery. The aim of further research is to verify if similar changes occur also in adipose tissue of humans subjected to bariatric surgery or having lipodystrophy.
